# Supplementary material for: Design and experiments of an automatic depth-adjusting double screw trencher and fertiliserning
Source: PLoS One. 2022 Dec 14;17(12):e0277824. doi: 10.1371/journal.pone.0277824 (PMC9750008; doi:10.1371/journal.pone.0277824)
Supplement: Attachment — Submitted filename: Response to Reviewers.docx [file pone.0277824.s001.docx]

Dear Editors and Reviewers

Thank you very much for your email regarding my manuscript (PONE-D-22-07555). We really thank you for giving us a valuable opportunity to improve our manuscript. According to your suggestion and referees’ comments, we have carefully revised the manuscript and addressed all the comments.

The revised version with changes in RED has been submitted electronically via the Web. Enclosed please find a letter detailing our revisions and reply to the comments. I hope the revised version is suitable for publication.

I am looking forward to hearing from you.

Best regards and wishes,

Zhiyu Song

**To Reviewer #1**

Comment 1: English editing.

**Response:** Thank you for your valuable advice.English editing has been completed.(Please see the revised version.).

Comment 2: Figure 7 is not clear.

**Response:** Thank you for your valuable advice. Figure 7 was regenerated in the data processing software with enlarged text and numeric fonts. (Please see the revised version.).

Comment 3: Please state the country of the Technical specification of quality evaluation for fertilizing machinery(NY/T 1003-2006).

**Response:** Thank you for your valuable suggestion.Technical specification of quality evaluation for fertilizing machinery(NY/T 1003-2006) is the Chinese standard.

Comment 4: Please give explanation whey the coefficient of variation shows a trend of decreasing as the speed of the spiral fertilizer

drain increases.

**Response:** Thank for your valuable suggestion. When the spiral fertilizer drain speed increases, the amount of fertilizer discharged also increases, and the increase in the amount of fertilizer discharged leads to uneven discharging, thus the coefficient of variation becomes larger.

Comment 5:  In introduction , no research papers using RSM were reviewed, specially in fertilization machines.

**Response:**Thank for your valuable suggestion. The research of RSM in fertilization machines has been added in the introduction.(Please see the revised version.).

Comment 6:  Provide φ (the filling factor of the spiral fertilizer drain).

**Response:**Thank for your valuable suggestion. φ is 0.25(the filling factor of the spiral fertilizer drain).

Comment 7: Does the fertilizer moisture content and its diameter had effect on coefficient of variation ? please give data or refences.

**Response:**Thank for your valuable suggestion.The moisture content of fertilizer has an effect on the coefficient of variation, for details please refer to the paper "Lu Fan. Design and experiment of cam top plate self-cleaning fertilizer apparatus[D]. Jiangxi Agricultural University", after the test, the diameter of fertilizer has no significant effect on the coefficient of variation.
