# Supplementary material for: Design and experiments of an automatic depth-adjusting double screw trencher and fertiliserning
Source: PLoS One. 2022 Dec 14;17(12):e0277824. doi: 10.1371/journal.pone.0277824 (PMC9750008; doi:10.1371/journal.pone.0277824)
Supplement: Attachment — Submitted filename: Response to Reviewers.docx [file pone.0277824.s002.docx]

Dear Editors and Reviewers

Thank you very much for your email regarding my manuscript (PONE-D-22-07555). We really thank you for giving us a valuable opportunity to improve our manuscript. According to your suggestion and referees’ comments, we have carefully revised the manuscript and addressed all the comments.

The revised version with changes in RED has been submitted electronically via the Web. Enclosed please find a letter detailing our revisions and reply to the comments. I hope the revised version is suitable for publication.

I am looking forward to hearing from you.

Best regards and wishes,

Zhiyu Song

**To Reviewer #1**

Comment 1: English editing.

**Response:** Thank you for your valuable advice.English editing has been completed.(Please see the revised version.).

Comment 2: Figure 7 is not clear.

**Response:** Thank you for your valuable advice. Figure 7 was regenerated in the data processing software with enlarged text and numeric fonts. (Please see the revised version.).

Comment 3: Please state the country of the Technical specification of quality evaluation for fertilizing machinery(NY/T 1003-2006).

**Response:** Thank you for your valuable suggestion.Technical specification of quality evaluation for fertilizing machinery(NY/T 1003-2006) is the Chinese standard.

Comment 4: Please give explanation whey the coefficient of variation shows a trend of decreasing as the speed of the spiral fertilizer

drain increases.

**Response:** Thank for your valuable suggestion. When the spiral fertilizer drain speed increases, the amount of fertilizer discharged also increases, and the increase in the amount of fertilizer discharged leads to uneven discharging, thus the coefficient of variation becomes larger.

Comment 5:  In introduction , no research papers using RSM were reviewed, specially in fertilization machines.

**Response:**Thank for your valuable suggestion. The research of RSM in fertilization machines has been added in the introduction.(Please see the revised version.).

Comment 6:  Provide φ (the filling factor of the spiral fertilizer drain).

**Response:**Thank for your valuable suggestion. φ is 0.25(the filling factor of the spiral fertilizer drain).

Comment 7: Does the fertilizer moisture content and its diameter had effect on coefficient of variation ? please give data or refences.

**Response:**Thank for your valuable suggestion.The moisture content of fertilizer has an effect on the coefficient of variation, for details please refer to the paper "Lu Fan. Design and experiment of cam top plate self-cleaning fertilizer apparatus[D]. Jiangxi Agricultural University", after the test, the diameter of fertilizer has no significant effect on the coefficient of variation.

**To Reviewer #2**

Comment 1: The different aspects of the existing machine from the literature or those made in the industry should be discussed and detailed.

**Response:**Thank for your valuable suggestion. Few domestic machines are available for fertilizer application specifically for tea plantations. Moreover, the available devices can perform a specific operation at a time such as trenching, fertilizer application, and mulching, and there is no provision for automatic depth adjustment, which leads to poor efficiency of fertilizer application, poor operation quality, and uneven fertilization . Globally, more advanced automated machinery and equipment have been developed specifically for fertilizer application in tea gardens. However, these are expensive, inflexible, unable to perform multitasking, and inconsistent with domestic tea garden planting standards. Therefore, this study proposes a novel design of an automatic depth-adjusting double-spiral trenching and fertilizer application machine, which can perform trenching, fertilizer application, and mulching operations, prevent soil accumulation, and improve fertilizer application quality and efficiency in tea gardens.(Please see the revised version.)

Comment 2: Does the existing machine have different sectoral application areas or can it be preferred for different sectoral applications in the industry? It should be discussed.

**Response:**Thank for your valuable suggestion.The fertilizing machine in this study is mainly used for fertilising tea plantations, but can also be used for fertilising crops with a planting row spacing greater than 1400mm and a fertiliser depth of less than 330mm.

Comment 3: Has a cost analysis been done in terms of economy? Details should be gave about the workpiece materials and cost information should be given or a comparison should be made with other machines in the industry related to the cost.

**Response:**Thank for your valuable suggestion.Because the spiral blade completes the cutting and slanting operation to the soil, and at the same time carries out the straight line ditching operation, it requires high strength and hardness of the material, so Cr12MoV with a thickness of 10mm is chosen as the material.The rest of the machined parts are made of stainless steel.

Comment 4: It is useful to give a figure of the manufacturing processes of the machine.

**Response:**Thank for your valuable suggestion.A figure of the manufacturing processes of the machine has been added, so please refer to Figure 6 of the paper.(Please see the revised version.)

Comment 5: How were the datas obtained with RSM verified? How many times were the tests repeated?

**Response:**Thank for your valuable suggestion.The trials were validated by field trials with three replications of each group.

Comment6: Conclusions section is too short. A general evaluation should be written and the results obtained should be detailed.

**Response:**Thank for your valuable suggestion.The conclusion section has been added.(Please see the revised version.)
